# Exploring Migraine Pathogenesis: Transcriptomic Insights and Pathway Analysis in Nitroglycerin-Induced Rat Model

**DOI:** 10.3390/cimb47040241

**Published:** 2025-03-30

**Authors:** Qiao-Wen Chen, Run-Tian Meng, Chih-Yuan Ko

**Affiliations:** 1 The School of Public Health, Fujian Medical University, Fuzhou 350122, China; 2 Department of Clinical Nutrition, The Second Affiliated Hospital of Fujian Medical University, Quanzhou 362000, China

**Keywords:** migraine, hypothalamus, transcriptomics, PI3K–Akt, RNA-seq

## Abstract

Migraine is a chronic neurovascular disease with unclear pathophysiological mechanisms. In this study, its pathogenic mechanisms were investigated through bioinformatics analysis of migraine-related pathways and key genes. Female Sprague Dawley rats were divided into control and migraine model groups. The control group received saline, while the migraine model group received nitroglycerin (NTG) to induce migraines over four weeks. Migraine-like behaviors were assessed within two hours following the final NTG injection. Genes of hypothalamus were identified using DESeq2. Gene ontology enrichment and KEGG pathway analyses were conducted, followed by the identification of hub genes based on protein interaction networks by using algorithms such as Closeness, Degree, and Maximum Neighborhood Component. Rats with NTG-induced migraine showed increased head scratching and cage climbing and a reduced sucrose preference. Transcriptome analysis revealed 1564 differentially expressed genes, with 1233 upregulated and 331 downregulated. Pathways linked to inflammation, PI3K–Akt signaling, and cytokine–cytokine receptor interactions were found to have enriched expression of several genes. Further protein interaction network analysis identified nine hub genes: *Alb*, *Tgfb1*, *Cd4*, *Ptprc*, *Itgb1*, *Icam1*, *Col1a1*, *Pxdn*, and *Itgad*. These findings suggest that migraine involves PI3K–Akt signaling and cytokine–cytokine receptor interactions, providing insights into molecular mechanisms and potential therapeutic targets. However, the study was limited by a small sample size and reliance on a single experimental model, which may constrain the clinical applicability of the findings.

## 1. Introduction

Migraine is a chronic neurovascular disorder characterized primarily by severe unilateral or bilateral throbbing headaches, often accompanied by symptoms indicative of autonomic nervous system dysfunction, such as nausea, vomiting, photophobia, and phonophobia. These episodes can last anywhere from several hours to several days [1]. Epidemiological studies have shown that migraine headaches are more prevalent in females [2], with a peak incidence especially during adolescence to childbearing and a gradual decline after menopause [3]. According to the 2019 Global Burden of Disease Report, headaches account for 5.4% of global years lived with disability (YLDs), with migraines comprising 88.2% of these, ranking second in YLDs [4], significantly contributing to socio-economic burdens [5].

The hypothalamus plays a central role in the initiation, maintenance, and resolution of migraine episodes [6]. Studies have demonstrated that bilateral cerebral blood flow in the hypothalamic region significantly increases during migraine attacks [7]. Moreover, abnormal functional connectivity between the hypothalamus and key pain-processing regions in the brainstem, such as the trigeminal nucleus, has been observed prior to migraine onset [8], indicating that the hypothalamus may initiate attacks by modulating these regions. During the interictal period, the functional connectivity between the hypothalamus and the limbic system is enhanced, whereas it is significantly reduced during the headache phase. This dynamic change may reflect impaired regulation of the limbic system by the hypothalamus, thereby contributing to migraine onset [9]. Premonitory symptoms such as mood fluctuations, fatigue, and yawning, along with autonomic dysfunctions like nausea and vomiting, also support the involvement of the hypothalamus in autonomic regulation in migraine patients [10]. In addition, the hypothalamus is involved in a complex neural network that includes the visual pathway and the trigeminovascular system. It receives nociceptive inputs via the trigeminohypothalamic tract and regulates pain processing in the spinal trigeminal nucleus. Disruption of this integrative network may contribute to the onset of migraine and related symptoms such as photophobia [11]. Therefore, further investigation into the role of the hypothalamus in migraine pathophysiology is crucial for understanding disease mechanisms and identifying new therapeutic targets.

Pathophysiological studies of migraine have primarily relied on animal models; however, due to the complex and multifactorial nature of the disease, no existing model can fully replicate all clinical features of migraine. Among these, the nitroglycerin (NTG)-induced model is widely adopted for its strong resemblance to the clinical presentation of migraine [12]. NTG, through the release of nitric oxide (NO), sensitizes the trigeminovascular system and promotes the release of endogenous calcitonin gene-related peptide from trigeminal ganglion neurons, inducing migraine-like pain and associated behaviors in rodents [13]. These manifestations align with the diagnostic criteria established by the International Headache Society [14]. Furthermore, clinical studies have demonstrated that intravenous administration of NO donors such as NTG can reliably provoke migraine-like headaches in susceptible patients [15]. The release of NO modulates various vasoactive amines, neuropeptides, and neurotrophic factors, thereby exacerbating trigeminovascular activation, nociceptive hypersensitivity, and enhancing the excitability of trigeminal neurons and glial cells. These effects contribute to the chronicity and recurrence of migraine episodes [16]. Given its strong clinical relevance and reproducibility, the NTG-induced model was employed in this study to investigate the underlying pathological mechanisms of migraine.

Transcriptomics research has been instrumental in uncovering molecular mechanisms underlying various neurovascular diseases. For instance, in cerebral hemorrhage models, transcriptomic analyses have pinpointed specific pathways and key regulatory genes associated with neuroinflammation, immune responses, and cell proliferation [17]. Similarly, in ischemic stroke, these analyses have identified critical regulatory genes involved in disease progression [18]. Building on these insights and methodologies, the present study focuses on examining changes in gene expression within the hypothalamus of female rats treated with NTG-induced migraine-like rodent model, hypothesizing that key alterations in genetic pathways could shed light on the molecular mechanisms driving migraine initiation and progression.

## 2. Materials and Methods

### 2.1. Experimental Animals

Female Sprague Dawley rats free of specific pathogens weighing 230–250 g were purchased from Beijing Viton Lever Biotechnology Co., Ltd. (Beijing, China). Rats were housed in a specific pathogen-free laboratory animal room with air circulation, maintained at 24–26 °C and 40–60% humidity, under a 12 h light/dark cycle, with free access to food and water. This study was approved by the Laboratory Animal Ethics Committee of the Second Affiliated Hospital of Fujian Medical University (No. 2022-117).

### 2.2. Migraine Model Animals

Subcutaneous injection of NTG (10 mg/kg; Henan Runhong, Xinzheng, China) at the nape of the neck is considered optimal for inducing migraine-like behaviors in rats [19]. Following a one-week acclimatization period, the experiment lasted for four weeks. Twelve rats were randomly assigned to two groups: a control group (Group C, *n* = 6) and a migraine model group (Group M, *n* = 6). Group C received a daily gavage of 0.9% saline (10 mL/kg), while Group M was subcutaneously injected with NTG at the nape of the neck on days 1, 7, 14, and 21 to induce migraine attacks. On all other days, Group M also received 0.9% saline by gavage [20].

### 2.3. Preparation of Tissues

All rats were anesthetized with an intraperitoneal injection of 2% sodium pentobarbital. Hypothalamus tissues were dissected according to the rat brain stereotaxic atlas and collected for transcriptomic sequencing.

### 2.4. Behavioral Experiments

**Figure 1:**
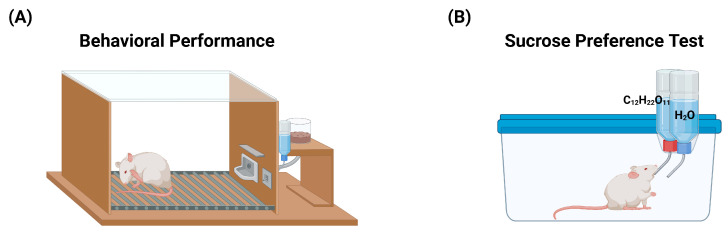
Behavioral performance (**A**) and sucrose preference test (**B**) equipment.

Behavioral changes were recorded to evaluate the success of migraine induction based on pain-related responses in rats [21]. Rats were individually placed in cages (16 × 18 × 29 cm) for a 5-min acclimation period. Following NTG or saline administration, rats were continuously recorded for 2 h using a camera (Epcbook, Shenzhen, China) to capture migraine-like behaviors (Figure 1A). Head scratching was defined as forepaw grooming of the lateral, temporal, or periorbital regions, while climbing behavior was defined as the use of forepaws to grasp the cage walls. All behavioral assessments were performed by investigators blinded to group assignments.

### 2.5. Sucrose Preference Test

During the initial sucrose adaptation phase, rats were provided with ad libitum access to two bottles, one containing tap water and the other a sucrose solution, in their home cages for 48 h, along with unrestricted access to food. Following this, the rats were moved to a testing apparatus, where they were again given free access to bottles with tap water and a sucrose solution, as well as ample food, for an additional 24 h to acclimate to the new environment. After this adaptation period, the rats underwent a 12 h fasting phase, during which they were deprived of both food and water. In the subsequent testing phase, each rat was placed individually in the testing apparatus with one pre-weighed bottle of sucrose solution and one pre-weighed bottle of tap water (Figure 1B). After 12 h, the bottles were reweighed to calculate the sucrose preference ratio, determined as (sucrose intake/total liquid intake) × 100%. Rats were excluded from the analysis if they exhibited no drinking behavior (non-drinkers), showed excessive consumption (intake greater than twice the average of other rats), or if control rats had a sucrose preference of less than 60% [22].

### 2.6. Transcriptome Sequencing Analysis

#### 2.6.1. RNA Library Construction and Sequencing

Total RNA was extracted using TRIzol reagent (Life Technologies, Carlsbad, CA, USA). RNA concentration and purity were measured with a NanoDrop 2000 spectrophotometer (Thermo Fisher Scientific, Wilmington, DE, USA), and RNA integrity was assessed using an RNA Nano 6000 Assay Kit on an Agilent Bioanalyzer 2100 system (Agilent Technologies, Santa Clara, CA, USA). mRNA was purified from total RNA using oligo magnetic beads. First-strand cDNA was synthesized, followed by second-strand cDNA synthesis. The blunt ends were generated by exonuclease and polymerase activity, and the 3′ ends of the DNA fragments were adenylated. These fragments were then ligated to NEBNext adapters with hairpin loop structures. The library fragments were purified using AMPure XP beads (Beckman Coulter, Beverly, MA, USA). Subsequently, 3 μL USER Enzyme (NEB, Ipswich, MA, USA) was added, and the sample was incubated at 37 °C for 15 min and denatured at 95 °C for 5 min before PCR. PCR amplification was performed using Phusion High-Fidelity DNA polymerase, universal PCR primers, and index (X) primers. The PCR products were purified using AMPure XP beads, and the library quality was assessed on an Agilent Bioanalyzer 2100 system. Sequencing was performed on an Illumina NovaSeq platform to generate 150 bp paired-end reads.

#### 2.6.2. Data Quality Control

Raw data (raw reads) of fastq format were first processed through in-house Perl scripts. In this step, sequences containing adapters, sequences containing ploy-N, and low-quality sequences were removed to obtain clean data. Metrics such as Q20, Q30, GC content, and sequence duplication level were calculated. All downstream analyses were based on high-quality clean data. Clean data were aligned to the reference genome using Hisat2, with only perfectly matched or single-mismatch sequences undergoing further analysis and annotation.

#### 2.6.3. Differentially Expressed Genes and Enrichment Analysis

Differential expression analysis between the groups was conducted using DESeq2v1.4.5, which employs a model based on the negative binomial distribution to determine differential expression in gene expression data. Genes with a DESeq2-derived *p*-value < 0.05 and fold change ≥ 1.5 were designated as differentially expressed genes (DEGs). An enrichment analysis of these DEGs in gene ontology (GO) and Kyoto Encyclopedia of Genes and Genomes (KEGG) pathways was performed using the clusterProfiler (3.8.1) software.

#### 2.6.4. Construction of Protein Interaction Networks and Screening Hub Genes

The STRING (https://stringdb.org, accessed on 1 March 2024) platform was used to construct the protein–protein interaction network (PPI) of genes (the species was set as “Rattus Norvegicus”, the minimum threshold was set to “maximum confidence” 0.4, and other parameters were set by default). The results were imported into Cytoscape (3.8.2) for visualization and analysis, and the pivotal genes were selected with the Cytohubba function based on three different scoring algorithms, i.e., Closeness, Degree, and Maximum Neighborhood Component (MNC), and the genes that were shared by these algorithms were selected as the hub genes.

### 2.7. Statistical Methods

SPSS 25.0 and GraphPad Prism 10 software were used for analysis and data visualization. Measurements are expressed as the mean ± standard error of the mean. *T*-tests were used for comparisons between two groups, while two-way ANOVA with Bonferroni post hoc tests were used for comparisons between groups. Differences were considered significant if the *p*-value was <0.05.

## 3. Results

### 3.1. Behavioral Observations

**Figure 2:**
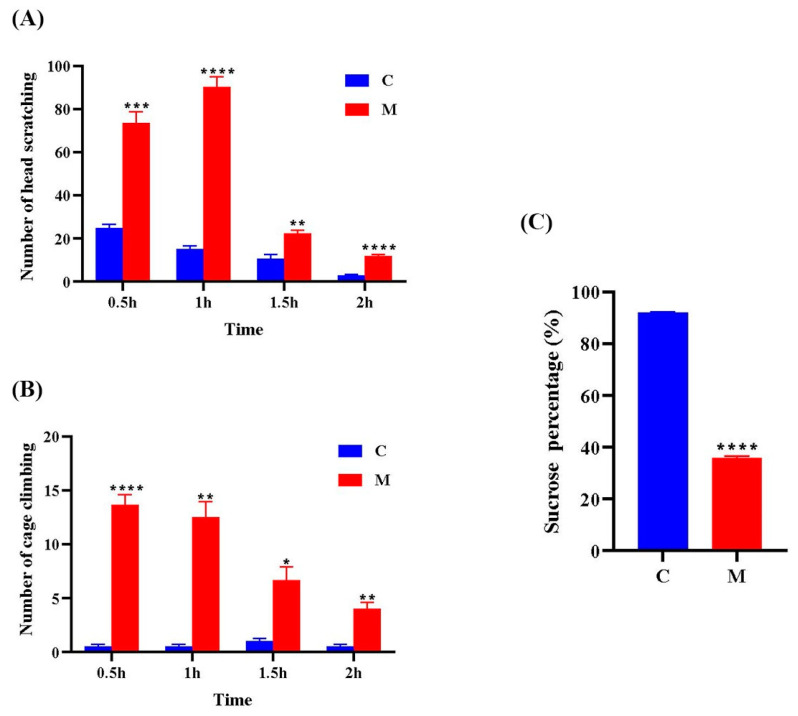
Changes in the number of head scratching (**A**) and cage climbing (**B**) behaviors at different time points (0.5 h, 1 h, 1.5 h, 2 h) after the last NTG injection in the control group and the migraine model group, as well as changes in water intake behavior (**C**). In each group (*n* = 6), data are expressed as mean ± SEM. C: control group; M: model group. * *p* < 0.05, ** *p* < 0.01, *** *p* < 0.001, **** *p* < 0.0001 vs. control.

Compared to Group C, Group M exhibited significantly increased head scratching ((Fgroup × time) = 65.34, *p* < 0.01) and cage climbing ((Fgroup × time) = 23.09, *p* < 0.05) across all time points (Figure 2A,B). In the sucrose preference test, Group M showed a markedly reduced sucrose preference rate compared to the control group (*p* < 0.0001) (Figure 2C). These results indicate a significant difference between Group C and Group M, confirming the successful induction of the migraine model.

### 3.2. Raw Data Quality Assessment

A total of 78.32 GB clean data were obtained from the transcriptome data, and the clean data of each sample reached 5.49 GB, and the percentage of Q30 bases was 97.86% and above. A sequence comparison of clean reads of each sample with the designated reference genome was performed, and the comparison efficiency ranged from 96.57% to 98.10%. The bases of the sequenced samples were of good quality and could be used for subsequent analysis of the transcriptome (Table 1).

### 3.3. Analysis of Transcriptional Patterns in the Migraine Model Group

**Figure 3:**
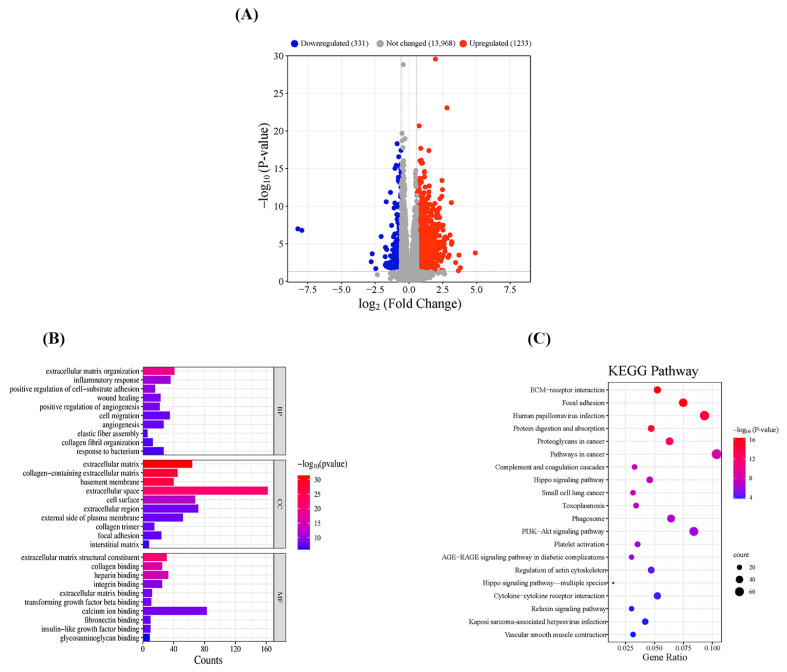
Transcriptional profiling of differentially expressed genes: (**A**) volcano map; (**B**) GO enrichment analysis map; (**C**) map of the top 20 KEGG pathways. A *p*-value less than 0.05 indicates statistical significance.

Compared with Group C, 1233 genes were upregulated and 331 genes were downregulated in Group M (Figure 3A). GO enrichment analysis showed that in terms of bioprocesses, the genes were mainly enriched in extracellular matrix organization, inflammatory response, etc.; in terms of cellular composition, they were mainly enriched in extracellular matrix, basement membrane, etc.; and in terms of molecular function, they were mainly enriched in extracellular matrix, collagen binding, etc. (Figure 3B). KEGG pathways showed the top 20 pathways with significant differences, mainly enriched in the PI3K–Akt and cytokine–cytokine receptor interaction signaling pathways (Figure 3C).

### 3.4. NTG Treatment Activates the PI3K–Akt Signaling Pathway in Migraine Rats

**Figure 4:**
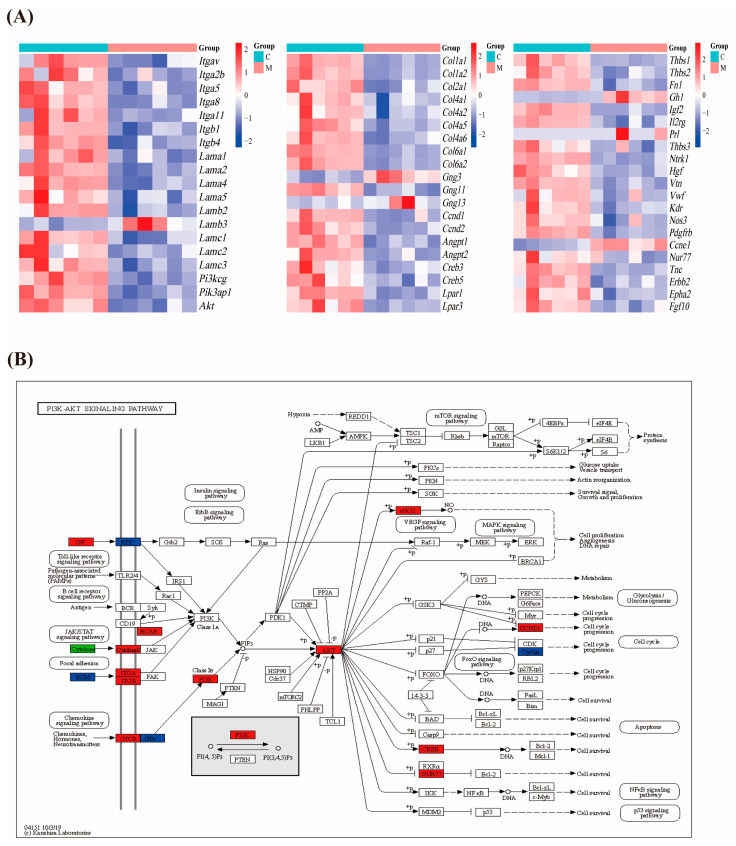
Activation of PI3K–Akt signaling pathway in migraine rats after NTG intervention: (**A**) heat map of differentially expressed genes; (**B**) visualization of the relationship between differentially expressed genes in the PI3K–Akt signaling pathway using the KEGG platform. Red color indicates upregulation, green color indicates downregulation, and blue color indicates both up- and downregulated genes.

The transcriptomics results indicated that NTG intervention upregulated *Itgav*, *Itga2b*, *Itga5*, *Itga8*, *Itga11*, *Itgb1*, *Itgb4*, *Lama1*, *Lama2*, *Lama4*, *Lama5*, *Lamb2*, *Lamc1*, *Lamc2*, *Lamc3*, *Pi3kcg*, *Pik3ap1*, *Akt*, *Col1a1*, *Col1a2*, *Col2a1*, *Col4a1*, *Col4a2*, *Col4a5*, *Col4a6*, *Col6a1*, *Col6a2*, *Gng11*, *Ccnd1*, *Ccnd2*, *Angpt1*, *Angpt2*, *Creb3*, *Creb5*, *Lpar1*, *Lpar3*, *Thbs1*, *Thbs2*, *Fn1*, *Igf2*, *Il2rg*, *Thbs3*, *Hgf*, *Vtn*, *Vwf*, *Kdr*, *Nos3*, *Pdgfrb*, *Nur77*, *Tnc*, *Erbb2*, *Epha2*, and *Fgf10*, while *Ccne1*, *Prl*, *Ntrk1*, *Gh1*, *Gng3*, *Gng13*, and *Lamb3* were downregulated (Figure 4A). Gene relationships are shown in Figure 4B.

### 3.5. NTG Treatment Activates Cytokine–Cytokine Receptor Interaction Signaling Pathway in Migraine Rats

**Figure 5:**
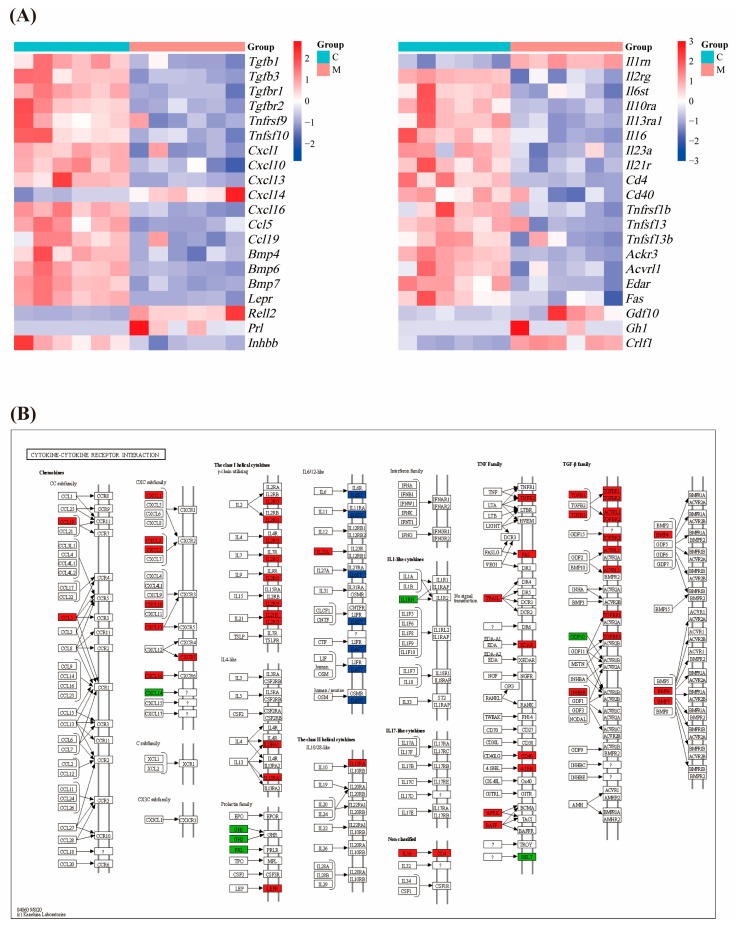
Activation of cytokine–cytokine interaction signaling pathway in migraine rats after NTG intervention: (**A**) heat map of differentially expressed genes; (**B**) visualization of the relationship between differentially expressed genes in the cytokine–cytokine interaction signaling pathway using the KEGG platform. Red color indicates upregulation, green color indicates downregulation, and blue color indicates both up- and downregulated genes.

The transcriptomics results showed that NTG intervention upregulated a large number of inflammation-related genes, including *Tgfb1*, *Tgfb3*, *Tgfbr1*, *Tgfbr2*, *Tnfrsf9*, *Tnfsf10*, *Cxcl1*, *Cxcl10*, *Cxcl13*, *Cxcl16*, *Ccl5*, *Ccl19*, *Bmp4*, *Bmp6*, *Bmp7*, *Il2rg*, *Il6st*, *Il10ra*, *Il13ra1*, *Il16*, *Il23a*, *Il21r*, *Cd4*, *Cd40*, *Tnfrsf1b*, *Tnfsf13*, *Tnfsf13b*, *Ackr3*, *Acvrl1*, *Edar*, *Fas*, *Gdf10*, *Crlf1*, *Lepr*, *Rell2*, and *Inhbb*, while *Cxcl14*, *Prl*, *Gh1*, *Il1rn*, *Crlf1*, *Rell2*, and *Gdf10* were downregulated (Figure 5A). The gene relationships are shown in Figure 5B.

### 3.6. PPI Network Construction and Screening of Hub Genes

**Figure 6:**
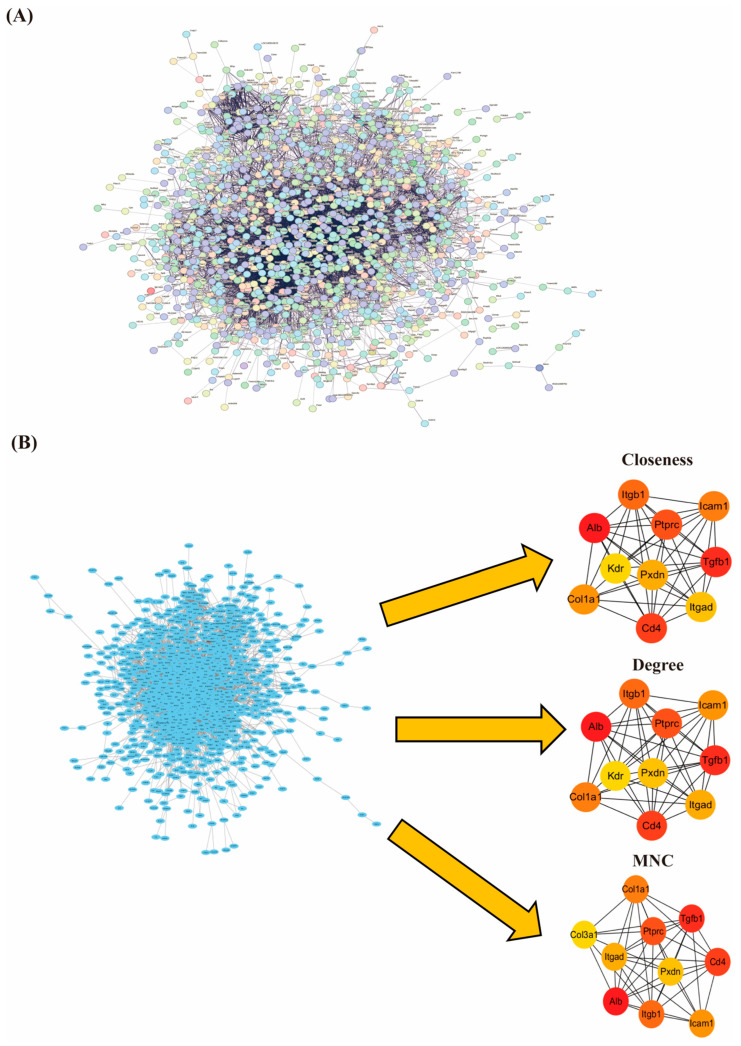
(**A**) Protein interactions map; (**B**) top 10 hub genes derived by each of the three algorithms Closeness, Degree, and MNC. The colors represent the importance of the genes, with red to yellow representing a gradual decrease in importance. MNC: Maximum Neighborhood Component.

The information of 1564 screened DEGs was imported into the STRING database, isolated nodes were removed, and the PPI network was constructed for DEGs with the condition that the interaction score was >0.4. The network included 1370 nodes and 9353 edges (Figure 6A). To further identify hub genes, we used the Cytohubba plugin and combined three different algorithms (Closeness, Degree, and MNC) to rate the top ten hub genes identified by each algorithm (Figure 6B). The results showed that serum albumin (*Alb*), transforming growth factor-β (*Tgfb1*), intercellular cell adhesion molecule-1 (*Icam-1*), cluster of differentiation 4 (*Cd4*), receptor tyrosine phosphatase type C (*Ptprc*), integrin beta-1 (*Itgb1*), collagen type I alpha-1 (*Col1a1*), peroxidasin (*Pxdn*), and integrin alpha-D (*Itgad*) were jointly identified as key pivotal genes in all three algorithms, suggesting that they may play a central role in migraine pathology.

## 4. Discussion

In this study, we used transcriptomics techniques to investigate the gene expression changes in the hypothalamus of NTG-induced migraine model rats. DEGs are involved in extracellular matrix composition, inflammatory response, angiogenesis, the PI3K–Akt signaling pathway, and the cytokine–cytokine receptor interaction pathway. Based on PPI analysis and Cytohubba analysis, *Alb*, *Tgfb1*, *Cd4*, *Ptprc*, *Itgb1*, *Icam1*, *Col1a1*, *Pxdn*, and *Itgad* were identified as nine hub genes. These results suggest that NTG-induced migraine is closely related to these key signaling pathways and targets, providing new insights into the molecular mechanisms of migraine.

Transcriptomic analysis identified the PI3K–Akt signaling pathway and cytokine–cytokine receptor interaction pathway as key contributors to the pathogenesis of migraine. Neurogenic inflammation is a critical mechanism underlying migraine attacks, and targeting this process is an effective strategy for promoting neurological recovery. The PI3K–Akt signaling pathway, a pivotal regulator of neurogenic inflammation, is widely expressed across various brain regions [23]. In the central nervous system, microglial activation plays a central role in neuroinflammatory responses. PI3K inhibitors can counteract the effects of TREM2 activation on LPS-induced neuroinflammation, thereby attenuating inflammatory responses [24]. Additionally, natural compounds such as flavonoids have been demonstrated to directly inhibit PI3K by binding to its ATP-binding site, consequently affecting Akt phosphorylation and activity [25]. In the NTG-induced migraine model, Chuanxiong Qingnao Granules significantly attenuated neuroinflammatory responses by inhibiting the PI3K–Akt signaling pathway [26], supporting the therapeutic potential of targeting this pathway in alleviating NTG-induced migraine. However, studies investigating the PI3K–Akt signaling pathway in NTG-induced migraine models in rats remain limited. These findings nonetheless suggest a promising new direction for migraine treatment. Regarding the cytokine–cytokine receptor interaction pathway, transcriptomic analysis revealed an upregulation of multiple inflammatory factors. Interleukin-16 (*IL-16*), a cytokine with chemotactic properties, has been shown to activate microglia and astrocytes through IL-16-CD4 signaling, thereby triggering inflammatory pain [27]. In addition, the expression of various chemokine family genes was upregulated, while the CXC motif chemokine ligand 14 (*Cxcl14*) was significantly downregulated. This suggests that *Cxcl14* may play a role in the proinflammatory phase, with its expression markedly reduced in an inflammatory environment [28]. Based on these transcriptomic findings, we propose that targeting neurogenic inflammation through the PI3K–Akt signaling pathway represents a promising therapeutic approach for the treatment of migraine.

Interestingly, transcriptomic analysis revealed a downregulation of prolactin (*Prl*) gene expression. Hormonal activity within the pituitary–hypothalamic axis plays a critical role in triggering migraine attacks, and prolactin, a 199-amino-acid peptide secreted by lactotroph cells in the anterior pituitary gland, is regulated by dopamine [29]. Current evidence on prolactin levels in migraine patients remains inconclusive. Some studies have reported significantly elevated serum PRL levels in migraine patients compared to those without headaches [30,31], while others have observed opposite findings [32]. This variability may be attributed to the complex hormonal interactions involved in migraine pathophysiology, highlighting the need for further investigation in future studies.

Through PPI analysis, this study identified nine hub genes closely associated with migraine, which may serve as potential biomarkers. Among them, *Alb*, *Tgfb1*, *Icam1*, *CD4*, and *Ptprc* are explicitly or potentially implicated in immune-inflammatory and oxidative stress mechanisms involved in migraine pathophysiology.

*Alb*, which encodes serum albumin, plays an antioxidant role by scavenging reactive oxygen and nitrogen species [33]. Studies have shown that serum Alb levels are significantly lower in migraine patients, particularly in females, potentially contributing to their reduced antioxidant capacity and higher migraine prevalence [34]. Furthermore, migraine often coexists with autoimmune disorders [35,36], and hypoalbuminemia has been correlated with autoimmune conditions [37], suggesting that Alb may be a key molecular link between immune dysregulation and migraine.

*Tgfb1* encodes transforming growth factor beta 1 (TGF-β1), a pleiotropic cytokine involved in immune modulation and inflammation [38]. Clinical studies have reported elevated TGF-β1 levels in the serum and cerebrospinal fluid of migraine patients [39,40], as well as persistently high platelet TGF-β1 levels even during interictal periods [41], indicating its relevance to migraine susceptibility.

*Icam1*, which encodes intercellular adhesion molecule-1 (ICAM1/CD54), facilitates leukocyte adhesion and transmigration during inflammation [42]. ICAM1 expression is upregulated during migraine attacks and may contribute to aseptic dural neurogenic inflammation [43]. Elevated levels of soluble ICAM1 have also been detected in the internal jugular vein within two hours after migraine onset [44].

*CD4* and *Ptprc* encode the T-cell surface glycoproteins CD4 and CD45, respectively, both of which are involved in T-cell activation and immune signal transduction [45,46].

In this study, transcriptomic analysis revealed significant downregulation of Alb and upregulation of *Tgfb1*, *Icam1*, *CD4*, and *Ptprc* in NTG-induced migraine model rats. These results suggest that oxidative stress, immune activation, and inflammation collectively contribute to the pathogenesis of migraine.

*Itgb1* and *Itgad* encode integrin β1 and integrin subunit CD11d, respectively. Both are key components of the extracellular matrix–receptor interaction pathway and are involved in the regulation of cell adhesion, migration, proliferation, and intracellular signaling [47,48]. These integrins may play synergistic roles in the neuroinflammatory and vascular abnormalities associated with migraine.

*Col1a1* and *Pxdn* encode a member of the collagen family and a heme peroxidase, respectively, both of which are implicated in cellular proliferation, metastasis, and oxidative stress responses [49,50]. Although current evidence does not directly link *Itgb1*, *Itgad*, *Col1a1*, and *Pxdn* to migraine, their functional relevance in inflammation and vascular remodeling suggests that they may contribute to migraine pathogenesis. Further investigation into the roles of these genes is warranted in view of the complex pathophysiology of migraine (Table 2).

This study has several limitations. First, the use of only female rats may limit the applicability of the findings to male populations, as sex differences are known to influence pain perception and immune function. Future studies should include both sexes to clarify sex-specific mechanisms in migraine. Second, the small sample size may reduce statistical power and the generalizability of the results. Although the observed differences reached statistical significance, the limited sample may not adequately represent broader biological variability. Third, this study utilized only the nitroglycerin-induced migraine model. While this model reproduces core features of migraine, it does not fully reflect the complex mechanisms involving genetic and environmental interactions or account for differences among migraine subtypes. Future research should include multiple models to improve translational relevance. Finally, further investigation of astrocytes and their interactions with neurons [51] is recommended to advance understanding of migraine pathophysiology.

## 5. Conclusions

In this study, we identified that NTG-induced migraine is associated with the PI3K–Akt signaling pathway, cytokines and their receptor interactions, and nine key hub genes (*Alb*, *Tgfb1*, *Cd4*, *Ptprc*, *Itgb1*, *Icam1*, *Col1a1*, *Pxdn*, and *Itgad*) through transcriptomics. These findings suggest that these factors may play a crucial role in the development and occurrence of migraine. Our study contributes to a better understanding of the complex pathomechanisms of migraine and suggests potential therapeutic targets for the future.

## Figures and Tables

**Table 1:** Statistics of sequencing data.

Sample	Clean Reads	Total Reads	Clean Bases	GC Content	≥Q30	Mapped Reads
C1	20,220,350	40,440,700	6,054,753,132	46.13%	97.87%	97.41%
C2	19,291,371	38,582,742	5,777,712,820	46.96%	98.35%	97.93%
C3	20,526,329	41,052,658	6,146,641,140	46.59%	97.97%	97.58%
C4	28,648,350	57,296,700	8,579,723,824	46.75%	98.37%	97.82%
C5	18,317,506	36,635,012	5,486,122,250	46.80%	98.36%	97.90%
C6	27,359,047	54,718,094	8,194,954,072	46.90%	98.16%	98.10%
M1	21,243,334	42,486,668	6,360,036,630	47.39%	97.86%	97.37%
M2	20,496,560	40,993,120	6,138,110,930	46.36%	98.69%	97.83%
M3	26,050,892	52,101,784	7,802,078,532	47.00%	98.41%	97.76%
M4	18,567,057	37,134,114	5,560,463,154	46.99%	98.27%	97.90%
M5	20,497,889	40,995,778	6,136,901,752	46.64%	97.87%	97.48%
M6	20,334,317	40,668,634	6,086,114,090	47.56%	99.12%	97.29%

Clean reads: the total number of pair-end reads in clean data; total reads: the number of clean reads with single-end; clean bases: the total number of bases in clean data; GC content: the GC content of clean data, i.e., the percentage of total bases accounted for by G and C bases; ≥Q30%: the percentage of bases with clean data quality value greater than or equal to 30; mapped reads: the number of reads compared to the reference genome and the percentage of them in the clean reads.

**Table 2:** Key genes associated with migraine.

Gene	Encoded Protein	Function	Clinical/Study Findings	log2FoldChange
*Alb*	Serum albumin	Effective removal of reactive oxygen species and active nitrogen.	Migraine patients, especially females, exhibit significantly reduced serum albumin levels compared to the general population [33].	−0.6639
*Tgfb1*	Transforming growth factor-β1 protein	Multifunctional cytokines are closely related to immune regulation and inflammatory responses.	TGF-β1 levels are significantly elevated in the serum and cerebrospinal fluid of individuals with migraine [39,40], and remain elevated in platelets even during headache-free intervals [41].	0.744684
*Icam-1*	Intercellular adhesion molecule-1 (ICAM1/CD54)	Mediates the migration and infiltration of white blood cells at the site of inflammation.	Soluble ICAM1 levels in internal jugular vein blood are transiently elevated within two hours following a migraine attack [44].	1.282695
*CD4*	CD4 glycoprotein	Involved in immune response and inflammation regulation.	No evidence currently links these genes to migraine.	1.613812
*Ptprc*	CD45 glycoprotein	Involved in T cell antigen receptor signal transduction.	No evidence currently links these genes to migraine.	1.176863
*Itgb1*	Integrin β1	Involved in extracellular matrix-cell receptor interactions.	No evidence currently links these genes to migraine.	0.613643
*Itgad*	Integrin subunit CD11d	Involved in immune responses such as leukocyte adhesion and migration.	No evidence currently links these genes to migraine.	1.604032
*Col1a1*	α1 chain of type I collagen	Involved in cell proliferation and metastasis.	No evidence currently links these genes to migraine.	2.617393
*Pxdn*	peroxidase	Antioxidant enzyme that catalyzes the oxidation of a wide range of substrates.	No evidence currently links these genes to migraine.	0.638802

## Data Availability

All data are available via the corresponding author.
